# Cystatin C as a Potential Blood Biomarker for Sarcoidosis: A Case Report

**DOI:** 10.7759/cureus.40304

**Published:** 2023-06-12

**Authors:** Shohei Fukunaga, Kenichi Itoga, Hirotaka Sonoda, Yuki Hoshino, Ryuichi Yoshimura, Masahiro Egawa, Takafumi Ito, Kazuaki Tanabe

**Affiliations:** 1 Nephrology, Shimane University Hospital, Izumo, JPN; 2 Internal Medicine, Shimane University Faculty of Medicine, Izumo, JPN

**Keywords:** lysozyme, soluble il-2 receptor, angiotensin-converting enzyme, cystatin c, sarcoidosis

## Abstract

Sarcoidosis is a multi-organ medical condition that is characterized by the formation of granulomas. We aimed to identify a correlation between each sarcoidosis blood biomarker and cystatin C (Cys-C) in sarcoidosis patients. We report a case of a 60-year-old man with sarcoidosis. The correlation between his Cys-C and each blood biomarker level and that between each blood biomarker and serum creatinine levels were determined using linear regression. Serum Cys-C correlated with each blood biomarker of sarcoidosis, while creatinine did not. These findings suggest that Cys-C is a potential blood biomarker for sarcoidosis.

## Introduction

Sarcoidosis is a multi-organ disease characterized by granuloma formation in various locations of the body. The clinical manifestations at onset and the subsequent clinical course vary. Although sarcoidosis frequently affects the hilar mediastinal lymph nodes, lungs, eyes, and skin, it can also affect most organs, including the nerves, muscles, heart, kidneys, bones, and digestive organs. Angiotensin-converting enzyme (ACE), soluble interleukin (IL)-2 receptor (sIL-2R), and lysozyme are representative blood biomarkers for sarcoidosis.

Although there are various markers for sarcoidosis, one blood biomarker alone is insufficient for establishing a sarcoidosis diagnosis. Hence, the identification of new blood biomarkers is warranted. Cystatin C (Cys-C) is a protease inhibitor that inhibits enzyme-induced cytoplasmic and tissue damage and bacterial and viral growth. Cys-C is used as a marker for early renal dysfunction. Recently, Cys-C has been reported to promote naive helper T-cells differentiation into inflammatory Th1/Th17 cells [1]. Sarcoidosis can lead to granuloma formation via a Th1-type cellular immune response, and Cys-C may be involved in its pathogenesis [2,3].

Here, we report a 60-year-old man with an unremarkable medical history who was diagnosed with sarcoidosis. We aimed to identify the correlation between the blood biomarkers of sarcoidosis and Cys-C in a patient with sarcoidosis.

## Case presentation

A 60-year-old Japanese man with serum creatinine (SCr) levels of 0.7 mg/dL was referred to our clinic for the management of renal dysfunction. The patient had no symptoms of arthritis, fatigue, weight loss, lymphadenopathy, or skin rash. He had an unremarkable urinalysis during a medical checkup in March 2021. However, a blood test performed in October 2021 showed a serum creatine (SCr) level of 1.65 mg/dL. An abdominal ultrasound revealed left hydronephrosis. Therefore, he visited the urologist at his local hospital, where he was diagnosed with a left ureteral stone. The stone was spontaneously resolved, and the hydronephrosis improved. No kidney stone analysis was performed. Although his hydronephrosis improved, he was referred to our department in January 2022 because of persistent renal dysfunction. The patient's medical history included hyperuricemia and hyperlipidemia. He regularly took ethyl omega-3 fatty acids, febuxostat, and atorvastatin. His dietary supplements also included sesamin and lactoferrin.

Blood tests revealed renal dysfunction (SCr: 1.34 mg/dL), a 2.6-fold discrepancy between estimated glomerular filtration rate (eGFR) and creat (43.1 mL/min/BSA and eGFR Cys-C (16.3); and hypercalcemia (calcium level: 11.1 mg/dL). Blood tests also revealed fractional excretion of calcium at 7.6% (calcium excretion was preserved, and there was no excessive intake of vitamins A and D) and low levels of parathyroid hormone (PTH) level (intact PTH 2.8 pg/mL). In addition, no elevation of PTH-related protein-intact level was not elevated (1.1 pmol/L), and high vitamin D levels (1-25 OH-D 124 pg/mL) were detected (Tables 1, 2).

**Table 1:** Urinalysis OB - occult blood; Plt - platelet; RBC - red blood cells; SG - specific gravity; WBC - white blood cells, U- urinary; FE - fractional excretion

Urinalysis	Result	Reference range (unit)
SG	1.007	1.010 - 1.030
Protein	-	-
OB	±	-
RBC	1-4	<4 (/HPF)
WBC	1-4	<4 (/HPF)
U-Na	26	No reference range (mmol/L)
U-Ca	21	No reference range (mg/dL)
U-Cre	33	No reference range (mmol/L)
FENa	4.0	No reference range (%)
FECa	7.6	No reference range (%)
U-Ca/Cr	0.43	No reference range

**Table 2:** Complete blood count and blood chemistry ACE - angiotensin converting enzyme inhibitor; Alb - albumin; ALT - alanine aminotransferase; ANA - antinuclear antibody; AST - aspartate aminotransferase; BUN - blood urea nitrogen; CA19-9 - carbohydrate antigen 19-9; CEA - carcinoembryonic antigen; CK - creatine kinase; Cr - creatinine; Cys-C - cystatine C; CRP - C-reactive protein; eGFR - estimated glomerular filtration rate; Eos - eosinophil; Hb - hemoglobin; HDL-C - high density lipoprotein cholesterol; Ht - hematocrit; intPTH - intact parathyroid hormone; LDH - lactate dehydrogenase; LDL - low density lipoprotein cholesterol; Lymph - lymphocytes; Neutro - neutrophils; Mono - monocyte; RBC - red blood cells; PTHrp-int - parathyroid hormone related protein intact; sIL2-2R - soluble interleukin-2 receptor; T-Cho - total cholesterol; TG - triglycerid; TP - total protein; UA - uric acid; VitD - vitamin D; WBC - white blood cells

Parameters	Result	Reference range (unit)
WBC	3990	3300-8600 (/μL)
Neutro	69.5	40.0-75.0 (%)
Eos	5.1	0.0-8.5 (%)
Baso	1	0.0-2.5 (%)
Mono	6.7	2.0-10.0 (%)
Lymph	17.7	16.5-49.5 (%)
RBC	412	435-555 ( x10^4^/μL)
Hb	12.4	13.7-16.8 (g/dL)
Ht	36.3	40.7-50.1 (%)
Plt	15.1	15.8-34.8 ( x10^4^/μL)
TP	6.5	6.6-8.1 (g/dL)
Alb	4.1	4.1-5.1 (g/dL)
AST	25	13-30 (U/L)
ALT	16	10-42 (U/L)
LDH	211	124-222 (U/L)
CK	103	59-248 (U/L)
BUN	24	8-20 (mg/dL)
Cr	1.34	0.65-1.07 (mg/dL)
Cys-C	3.27	0.63-0.95 (mg/dL)
eGFR creat	43.1	> 60 (mL/min/BSA)
eGFR Cys-C	16.3	> 60 (mL/min/BSA)
T-Cho	231	142-248 (mg/dL)
TG	315	40-149 (mg/dL)
HDL-C	42	40-90 (mg/dL)
LDL-C	104	65-139 (mg/dL)
Na	140	138-145 (mmol/L)
K	3.8	3.6-4.8 (mmol/L)
Cl	103	101-108 (mmol/L)
Ca	11.1	8.8-10.1 (mg/dL)
P	2.9	2.7-4.6 (md/dL)
CRP	0.1	< 0.14 (mg/dL)
CA19-9	14.1	< 37 (U/mL)
CEA	3.3	< 5.0 (ng/mL)
ANA	<40	< 40
1-25VitD	124	20-60 (pg/mL)
intPTH	2.8	10-65 (pg/mL)
PTHrp-int	1.1	< 1.1 (pmol/L)
ACE	35.8	8.3-21.4 (U/L)
sIL-2R	3178	145-519 U/mL
Lysozyme	33.6	4.2-11.5 (μg/L)

**Figure 1:**
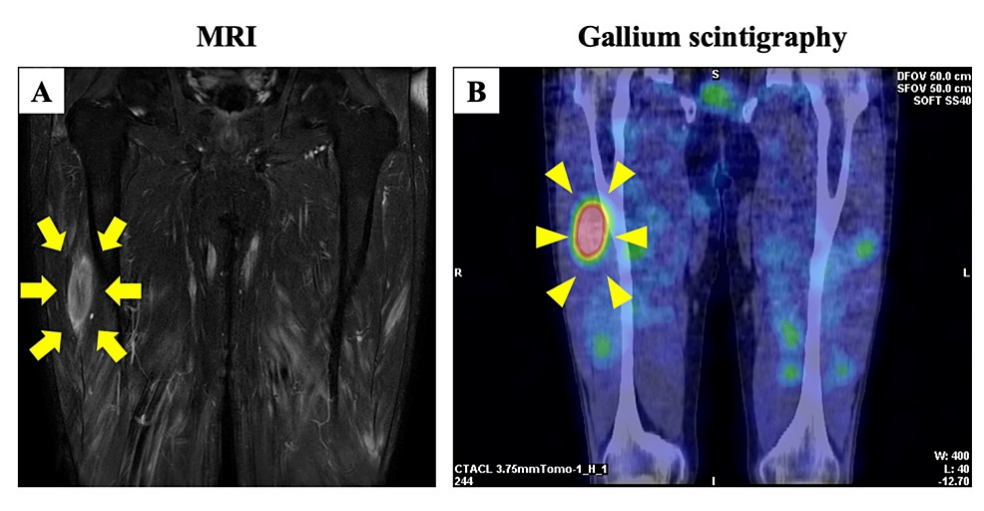
MRI (T2WI) and gallium scintigraphy (A) MRI (T2WI) shows the three stripe and dark star signs in the granuloma in the right thigh muscle layer (yellow arrow). (B) Gallium scintigraphy reveals accumulation in the same area (yellow arrow head).

**Figure 2:**
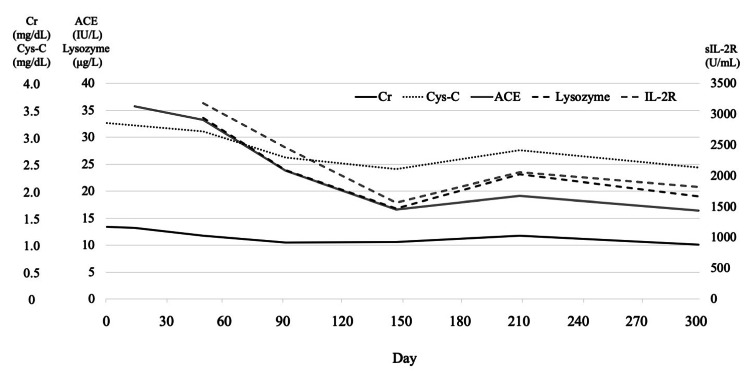
Clinical course of the patient ACE - angiotensin converting enzyme inhibitor; Cr - creatinine; Cys-C - cystatin C; sIL-2R - soluble interleukin-2 receptor

Chest radiography and computed tomography findings were negative for signs of tuberculosis, and no bilateral hilar lymphadenopathy was observed. Magnetic resonance imaging (MRI) (T2WI) showed the three stripe sign and dark star signs in a granuloma in the right thigh muscle layer (Figure 1A). Gallium scintigraphy revealed accumulation in the same area (Figure 1B). Percutaneous needle biopsy revealed non-necrotizing granuloma formation, and muscular sarcoidosis was diagnosed. Echocardiography and fundoscopy showed no remarkable change, and the patient had no subjective symptoms. Therefore, the patient was treated conservatively without administration of corticosteroids or immunosuppressive drugs. He has not taken any oral supplements containing vitamin D. He preferred eating mushrooms, a food rich in vitamin D. Therefore, the patient was instructed to avoid food containing high vitamin D levels. The patient was closely monitored (Figure 2).

The eGFR Cys-C and eGFR creat were compared using the paired t-test, and correlations between Cys-C and sarcoidosis blood biomarkers (ACE, sIL-2R, and lysozyme) were assessed using linear regression analysis. The correlation between SCr and each blood biomarker of sarcoidosis was evaluated using the same method. Prism version 7.0 (GraphPad Software Inc., San Diego, CA) was used for statistical analysis. A p-value of <0.05 was considered statistically significant. 

**Figure 3:**
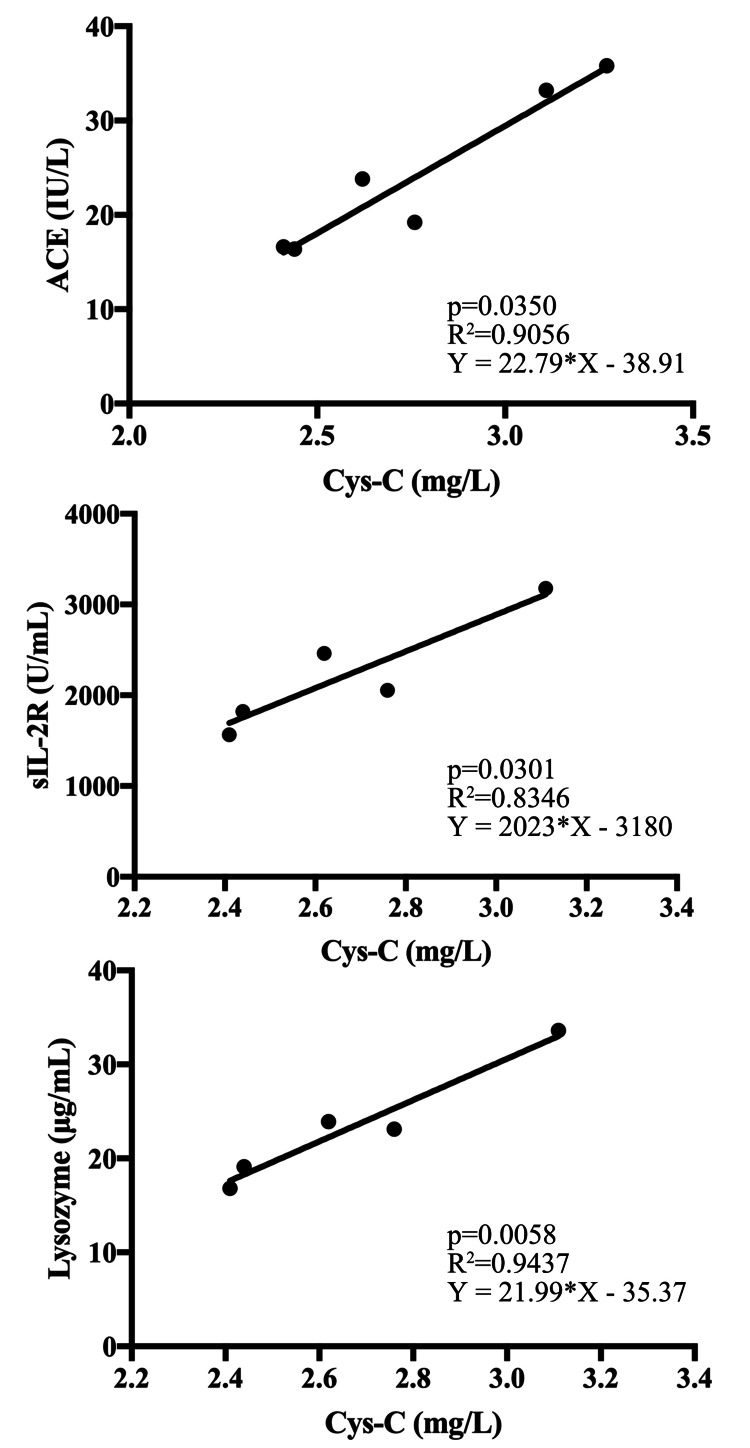
Relationship between cystatin C and ACE, sIL-2R, and lysozyme A correlation is observed between Cys-C and ACE, sIL-2R, and lysozyme. ACE - angiotensin-converting enzyme; Cys-C - cystatin C; sIL-2R - soluble IL-2 receptor

**Figure 4:**
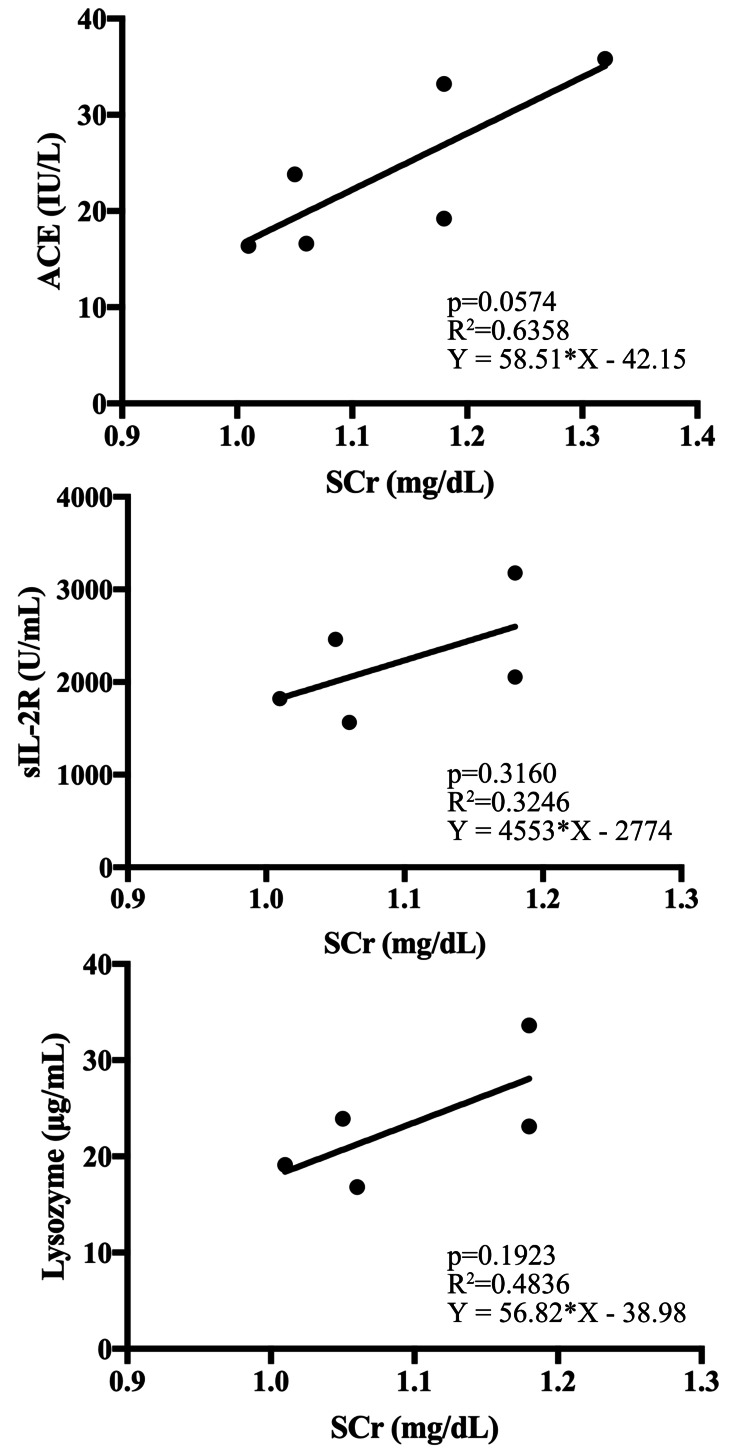
Relationship between serum creatinine and ACE, sIL-2R, and lysozyme No correlation is observed between serum creatinine and ACE, sIL-2R, and lysozyme. ACE - angiotensin-converting enzyme; SCr - serum creatinine; sIL-2R - soluble IL-2 receptor

eGFR Cys-C was significantly lower than eGFR creat (21.1 ± 1.5 versus 51.9 ±2.3; p<0.0001). A correlation was observed between Cys-C and ACE, IL-2R, and lysozyme (Figure 3) but not between the blood biomarkers and SCr (Figure 4).

As a case report, the study was exempt from ethics approval. The patient provided informed consent.

## Discussion

Although the cause of sarcoidosis is unknown, it is thought that, in susceptible individuals, there is a Th1-type cellular immune response (type IV allergic reaction) to the antigen that causes the disease, resulting in granuloma formation in various organs of the body [2,3].

ACE, sIL-2R, and lysozyme are included in the criteria for the clinical diagnosis of sarcoidosis in Japan [4]. In patients with sarcoidosis, ACE is thought to be produced by monocytic cells, including all epithelioid cells within the granuloma tissue. Therefore, the serum ACE level reflects the total amount of granuloma [5]. Although the detection of serum ACE levels has a specificity of 90% for sarcoidosis diagnosis, the sensitivity is 57%, which is insufficient to make a definite diagnosis [6]. sIL-2R is released from activated T cells and is a sensitive indicator of T cell activation [7]. In patients with sarcoidosis, the blood sIL-2R levels are elevated [8]. Although the positivity rate is >60%, it should be noted that sIL-2R levels are also elevated in cases of malignant lymphomas and collagen diseases. Lysozyme is an enzyme produced by monocytes and macrophages with a reported sensitivity of 79.1% [9]. However, its specificity is low because its level has been reported to be elevated by multiple diseases, including those similar to sarcoidoses, such as tuberculosis, silicosis, asbestosis, and berylliosis: these diseases are characterized by granulomatous inflammation. This is why one blood biomarker alone is insufficient for the diagnosis of sarcoidosis, and multiple blood biomarker levels must be determined. Therefore, the establishment of new blood biomarkers is desired.

Cys-C is an indicator of renal function that is independent of muscle mass. Although Cys-C levels rarely fluctuate in conditions other than renal dysfunction, they can be elevated in patients with melanoma, rectal cancer, and corticosteroid use and decreased in those with HIV infection and cyclosporine use [10-12]. In our setting, the aforementioned diseases were not present, and the medications were not being used by our patient. However, eGFR Cys-C was significantly lower than eGFR Creat. Furthermore, the biomarkers (ACE, sIL-2R, and lysozyme) were correlated with Cys-C but not with SCr. Therefore, Cys-C may be a potential blood biomarker for sarcoidosis. Cys-C can promote the differentiation of naïve helper T cells into inflammatory Th1/Th17 [1]. In patients with sarcoidosis, a Th1-type cellular immune response occurs, and Cys-C may be involved in its pathogenesis. Th1-type cell promotes the activation of mononuclear cells. Furthermore, Th17-type cell recruits and activates neutrophils. However, no abnormalities in the differential count of leukocytes were observed in this patient. Eosinophilia has been reported in 16% and monocytosis in 12% of patients with sarcoidosis [13]. Additionally, abnormalities in the differential count of leukocytes are only found in some cases. Therefore, evaluating the activated state of Th1-type cellular and Th17-type cells only based on the differential count of leukocytes is difficult. In this case, Th1 and Th17-type cells were not directly evaluated. Therefore, the relationship between cystatin C and Th1 and Th17 in sarcoidosis cases should be further investigated. Further studies are needed to understand the involvement of Cys-C in the pathogenesis of sarcoidosis.

## Conclusions

This is the first report on a correlation between the three biomarkers for sarcoidosis and Cys-C. The mechanism by which cystatin C is elevated in patients with sarcoidosis was not established in this report, necessitating further elucidation. We reported Cys-C as a potential blood biomarker for sarcoidosis. Since this is a report of a single patient, more reports should be accumulated and reviewed.
